# Anti-Myeloperoxidase Antibodies Associate with Future Proliferative Lupus Nephritis

**DOI:** 10.1155/2017/1872846

**Published:** 2017-12-24

**Authors:** S. W. Olson, J. J. Lee, M. Poirier, D. J. Little, L. K. Prince, T. P. Baker, J. D. Edison, K. C. Abbott

**Affiliations:** ^1^Walter Reed National Military Medical Center, 8901 Wisconsin Avenue, Bethesda, MD 20889, USA; ^2^Naval Medical Center San Diego, 34800 Bob Wilson Drive, San Diego, CA 92134, USA; ^3^San Antonio Military Medical Center, 3551 Roger Brooke Dr., Fort Sam Houston, TX 78234, USA; ^4^The National Institute of Diabetes and Digestive and Kidney Diseases (NIDDK), NIH, 6707 Democracy Blvd, Bethesda, MD 20892, USA

## Abstract

**Background:**

The subclinical pathophysiology of proliferative lupus nephritis (PLN) has not been fully elucidated. Myeloperoxidase anti-neutrophil cytoplasmic antibody (MPO-ANCA) is associated with PLN, but prediagnostic levels have not been reported.

**Methods:**

We performed a retrospective case-control Department of Defense Serum Repository (DoDSR) study comparing MPO-ANCA levels in longitudinal prediagnostic serum samples for 23 biopsy confirmed proliferative lupus nephritis (PLN) patients to DoDSR identified age, sex, race, and age of serum matched healthy and SLE without LN disease controls. We also compared the temporal relationship of MPO-ANCA to anti-double stranded DNA antibodies (dsDNAab).

**Results:**

A greater proportion of PLN patients had prediagnostic MPO-ANCA levels above ≥3 U/mL and ≥6 U/mL compared to SLE without LN (91% versus 43%, *p* < 0.001; 57% versus 5%, *p* < 0.001, resp.). In subgroup analysis, the MPO-ANCA threshold of ≥3 U/mL was significant at <1 year (88% versus 39%, *p* = 0.007) and 1–4 years (87% versus 38%, *p* = 0.009) prior to diagnosis. Statistically significant subclinical MPO-ANCA levels (≥3 U/mL) occurred prior to statistically significant dsDNAab ≥ 3 IU/ml (89% versus 11%, *p* = 0.003).

**Conclusions:**

Subclinical MPO-ANCA levels could distinguish future PLN from SLE without LN. MPO-ANCA manifests prior to clinical disease and subclinical dsDNAab to suggest that it may contribute directly to PLN pathogenicity.

## 1. Introduction

Systemic Lupus Erythematosus (SLE) is a morbid autoimmune disease with multisystem organ involvement [1, 2]. Lupus Nephritis (LN) occurs in approximately half of SLE patients. The pathogenesis of SLE and LN is much better understood but not yet fully elucidated [3]. More than one mechanism of disease, each with multiple required deficits, may exist. Numerous autoantibodies are associated with both SLE and LN to include antineutrophil cytoplasmic antibodies (ANCA) [4, 5]. Perinuclear antineutrophil cytoplasmic antibodies (pANCA) and specifically anti-myeloperoxidase antibody (MPO-ANCA) are the predominant ANCA reported [5]. Simultaneous overt clinical manifestation of both ANCA associated vasculitis (AAV) and SLE has been reported [6–9]. Even without clinical evidence of AAV, ANCA is positive in 16%–42% of SLE patients [10–15]. A variable number of LN patients may have caused this wide range. ANCA is positive in 37%–53% of LN patients and more common in LN patients than SLE patients without LN [16–19]. ANCA is also more strongly associated with diffuse proliferative LN (PLN) than other types of LN, as well as more common in crescentic PLN than PLN without crescents [19, 20]. While the contribution of ANCA to AAV has been well described, it is unclear if ANCA directly contributes to LN pathogenesis or is simply a passive marker of disease [21–23]. SLE relapse is associated with positive ANCA levels [24]. But only presence, and not absolute level of ANCA, has been correlated with disease severity. No previous studies have evaluated prediagnostic ANCA levels or their temporal relationship with other biomarkers to provide insight into subclinical SLE and LN pathophysiology. Arbuckle et al. described the evolution of prediagnostic autoantibodies in a general SLE population but did not evaluate MPO-ANCA [22]. We recently reported that dsDNAab more frequently became elevated before PLN than before SLE without LN [23]. Subclinical ANCA levels were measured in the same Department of Defense Serum Repository (DoDSR) serum samples. We hypothesized that ANCA would be elevated in a larger percent of PLN subjects prior to diagnosis than SLE patients without LN and healthy controls. Based on our previous discovery that ANCA is present prior to anti-GBM antibody in anti-GBM disease, we also hypothesized that ANCA levels would be elevated prior to both dsDNAab and CRP to suggest a direct contribution to PLN pathophysiology [24, 25].

## 2. Materials and Methods

We performed a retrospective case-control serum bank study comparing MPO-ANCA and PR3-ANCA levels years prior to PLN diagnosis to matched healthy and SLE without LN disease controls. We previously reported preclinical dsDNAab and CRP levels for these same patients and serum samples.

As described previously, we identified 23 cases of biopsy proven PLN (WHO class III or WHO class IV) from the Walter Reed Army Medical Center renal biopsy database from 1993 to 2009. A comprehensive electronic data base review was performed for each PLN case to populate a clinical background data collection sheet. None of the cases had medications in their record that were associated with drug-induced lupus.

The Department of Defense Serum Repository (DoDSR) identified 23 age, sex, race, and age of serum sample matched healthy and 21 SLE without LN disease controls [23]. Each disease control had at least one hospitalization or three outpatient ICD-9 codes for SLE (711.0) without an ICD-9 code for lupus nephritis (583.81) or any other urinary abnormalities to suggest undiagnosed LN. The more rigorous selection criteria minimized the chance of including false positive cases that were erroneously coded during an ultimately negative SLE workup. The DoDSR also provided a list of all other ICD-9 codes for each matching disease control to document comorbidities. Walter Reed specific SLE without LN disease controls would not have been matched effectively for age, sex, race, and age of serum sample. This could have introduced insurmountable confounders into the final data set.

The DoDSR then pulled the oldest, the second most recent, and the most recent 0.5 mL serum samples prior to PLN or SLE without LN diagnosis and sent them to Quest Diagnostics Nichols Institute (Chantilly, VA).

### 2.1. Laboratory Assays

#### 2.1.1. Measurement of MPO-ANCA

Quantitative measurement of MPO-ANCA antibody serum concentration was performed using the Varelisa™ MPO-ANCA EIA kit (Phadia GmbH, Freiburg, Germany). Serum aliquots from the subjects were diluted 1 : 101 with sample diluent and run in duplicate. Microwells were precoated with purified human MPO. 100 microliters each of calibrators (0, 3, 7, 16, 40, and 100 U/ml), controls, and diluted subject serum samples were dispensed into the wells, and the assay was performed as described in the instructions for use. The secondary antibody was a horseradish peroxidase-labeled anti-human IgG conjugate, and detection was achieved using TMB stopped with phosphoric acid solution. Absorbance was determined at 450 nm. The results are reported in U/ml, with a measuring range of 1.0 to 100 U/ml, and a detection limit of 1.0 U/ml. A clinically negative result was indicated by <6.0 U/ml. Intra-assay variability was 4.8 to 9.5 CV, and interassay variability was 3.5 to 10.7 CV over the range of the standards. The frequency distribution in 432 healthy subjects was 1.5 U/ml (95th percentile, 3.4 U/ml).

#### 2.1.2. Measurement of PR3-ANCA

Quantitative measurement of PR3-ANCA antibody serum concentration was performed using the Varelisa™ PR3 ANCA EIA kit (Phadia GmbH, Freiburg, Germany). Serum aliquots from the subjects were diluted 1 : 101 with sample diluent and run in duplicate. The microwells were precoated with purified human neutrophil PR3. 100 microliters each of calibrators (0, 3, 7, 16, 40, and 100 U/ml), controls, and diluted subject serum samples were dispensed into the wells, and the assay was performed as described in the instructions for use. The secondary antibody was a horseradish peroxidase-labeled anti-human IgG conjugate, and detection was achieved using TMB stopped with phosphoric acid solution. Absorbance was determined at 450 nm. The results are reported in U/ml, with a measuring range of 0.5 to 100 U/ml and a detection limit of 0.5 U/ml. A clinically negative result was indicated by <6.0 U/ml. Intra-assay variability was 4.8 to 5.9% CV, and interassay variability was 2.4 to 9.3% CV over the range of the standards. The frequency distribution in 432 healthy subjects was 0.7 U/ml (95th percentile, 1.2 U/ml). For all three assays, the results were rounded to and reported in whole numbers.

### 2.2. Statistical Analysis

The percent of PLN subjects with MPO-ANCA above selected threshold values prior to diagnosis was compared to healthy and SLE without LN disease controls using the Fisher exact probability test. Odds ratios and 95% confidence intervals were also calculated. The same statistical analysis was used for all secondary outcomes and subgroup analysis. Conditional logistic regression and ROC curves were performed using STATA 12.0. Absolute MPO-ANCA change per year was calculated by dividing the difference between last MPO-ANCA (MPO-ANCA*l*) minus the index MPO-ANCA (MPO-ANCA*i*) by the difference in days (*T*) between the two samples (*Tl* − *Ti*) and multiplying the total by 365 days/year (MPO-ANCA*l* − MPO-ANCA*i*)/(*Tl* − *Ti*)*∗*365. Infinite odds ratio values were estimated by adding 1 to the numerator (if 0 controls were positive) or denominator (if all study participants were positive) of both the disease and control groups.

The DoDSR was not able to assign a matching disease control for one case. Serum samples for a second disease control were accidently destroyed at Quest leaving two less disease control subjects for analysis of the entire time period prior to diagnosis. Not all subjects had samples available for each subgroup time period. If multiple serum samples were present for a subject in a specific subgroup analysis time period, the highest antibody level dictated group assignment. Only 20 study subjects had multiple serum samples for evaluation of change in MPO-ANCA levels over time. Antecedent rise in MPO-ANCA versus dsDNAab or CRP was established only for patients with a clear initial biomarker elevation. If both became elevated in the same sample or if neither was elevated in any sample prior to diagnosis, there was no antecedent elevation determined.

This study was approved by the Human Use Committee at Walter Reed National Military Medical Center and informed consent was waived.

## 3. Results

### 3.1. Demographics

As previously reported, this study population consisted of predominantly African American females less than 40 years old. Joint, hematologic, and dermatologic involvement was most common (Table 1). Only three of the study patients had ANCA labs at diagnosis and all were negative. The majority of PLN subjects had an elevated dsDNAab, hematuria and/or proteinuria, and WHO class IV LN on biopsy at diagnosis (Table 1). There was no statistically significant difference of joint, skin, cardiac, pulmonary, or hematologic involvement between the PLN group and the SLE without LN disease control group (Table 1).

### 3.2. MPO-ANCA and PR3-ANCA

A greater percentage of PLN cases had an elevated MPO-ANCA level (≥6 U/mL) compared to both matched healthy and SLE without LN disease controls at any time prior to diagnosis (57% versus 0%, *p* < 0.001; 57% versus 5%, *p* < 0.001, resp.) and in the subgroup less than one year prior to diagnosis (59% versus 0%, <0.001; 59% versus 8%, *p* = 0.006, resp.). There was no significant difference found in the subgroups of 1–4 years and >4 years prior to diagnosis (Table 2). More PLN patients had an MPO-ANCA level ≥ 3 U/mL compared to matched healthy and disease controls at any time (91% versus 26%, *p* < 0.001; 91% versus 43%, *p* < 0.001, resp.), less than one year prior to diagnosis (88% versus 19%, *p* < 0.001; 88% versus 39%, *p* = 0.007, resp.), 1–4 years (87% versus 13%, *p* < 0.001; 87% versus 38%, *p* = 0.009, resp.), and >4 years (69% versus 25%, *p* = 0.03; 69% versus 44%, *p* = 0.29, resp.) prior to diagnosis (Table 2). Conditional logistic regression demonstrated that each incremental U/ml rise of MPO-ANCA increased the odds of future PLN diagnosis compared to both healthy controls and SLE without LN disease controls (OR 9.1 [3.4–24.5], *p* < 0.001; OR 1.5 [1.2–1.8], *p* < 0.001, resp.; Table 3).

**Figure 1:**
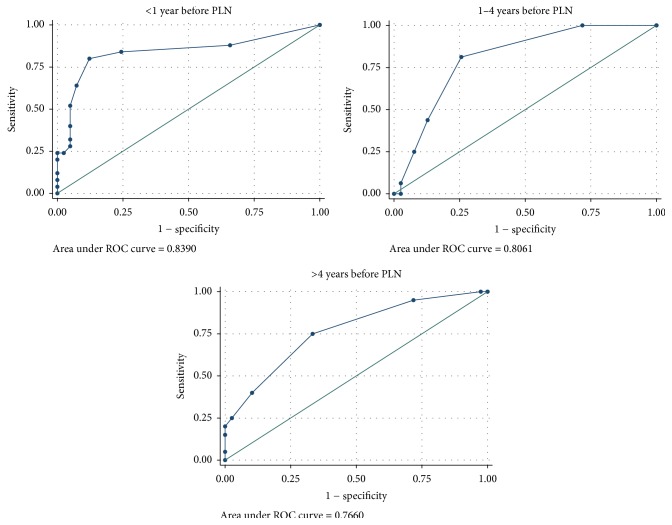
MPO-ANCA receiver operating curves for PLN cases versus healthy controls for <1 year, 1–4 years, and >4 years before PLN diagnosis.

**Figure 2:**
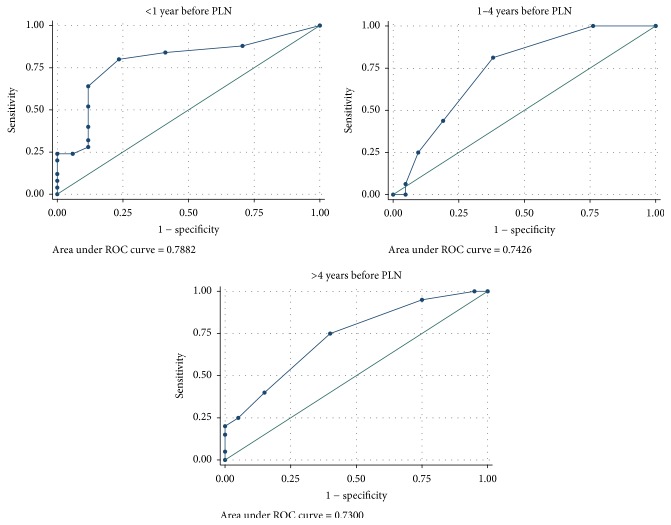
MPO-ANCA receiver operating curves for PLN cases versus SLE without LN disease controls for <1 year, 1–4 years, and >4 years before PLN diagnosis.

The MPO-ANCA ROC area under the curve was greater than 0.7 at less than 1 year, 1–4 years, and greater than 4 years for analysis of PLN cases with both healthy controls (Figure 1) and SLE without LN disease controls (Figure 2).

Neither the PLN cases, nor the disease and healthy controls had an elevated PR3-ANCA level in any serum sample. A greater percentage of PLN cases had a PR3-ANCA level ≥ 2 U/ml compared to both matched healthy and disease controls at any time prior to diagnosis (30% versus 4%, *p* = 0.04 for both).

### 3.3. Time Course of Antibody Development

In the PLN patients, MPO-ANCA became elevated a median of 139 days (25%, 75%; 63, 258 days) prior to diagnosis. MPO-ANCA was ≥3 U/mL a median of 5.75 years prior to diagnosis (25%, 75%; 1.3, 7.5 years) which is an underestimation because this level was present oldest index sample in 81% of the patients. PR3-ANCA ≥ 2 U/mL was only present in serum samples with a concurrent elevated MPO-ANCA.

A greater percentage of PLN cases had a rise in MPO-ANCA over time in comparison to both healthy and SLE without LN disease controls (70% versus 10%, *p* = 0.001; 70% versus 16%, *p* < 0.001, resp.; Table 4). Only PLN cases had a MPO-ANCA rate of rise greater than 0.5 U/mL/year (45% versus 0%, *p* = 0.001) or an absolute rise greater than 3 U/mL (30% versus 0%, *p* = 0.02; Table 4). In addition, a MPO-ANCA of ≥3 that rose more than 1 U/ml over time was highly specific for PLN (45% versus 0%, *p* = 0.001)

### 3.4. MPO-ANCA versus dsDNAab and CRP

The lowest statistically significant subclinical MPO-ANCA level (≥3 U/mL), 50% of the threshold for clinical disease, was present prior to the lowest statistically significant subclinical dsDNAab level (≥3 U/mL), as well as the dsDNAab level that is 50% of the threshold for clinical disease (≥5 U/mL) when it was possible to ascertain an antecedent antibody (89% versus 11%, *p* = 0.003; 100% versus 0%, *p* < 0.001, resp.; Table 5). It was not possible to determine temporal relationship of antibodies in cases in which both antibodies crossed threshold values in the same serum sample or did not cross the threshold in any samples.

There was no association between elevated dsDNAab and elevated MPO-ANCA (Supplemental Table  1). DsDNA antibody was as high as 640 IU/ml in the setting of a low normal MPO-ANCA level ≤ 3 U/mL. MPO-ANCA was as elevated as high as 16 in the setting of a low normal dsDNA ≤ 5 U/mL.

More PLN patients had an MPO-ANCA level ≥ 3 U/mL prior to an elevated CRP of >0.8 mg/dL (100% versus 0%, *p* < 0.001)

## 4. Discussion

In addition to SLE, MPO-ANCA has been described at diagnosis in subpopulations of multiple autoimmune and inflammatory diseases to include anti-GBM disease, IgA nephropathy, and inflammatory bowel disease [24–30]. It is not clear if MPO-ANCA contributes directly upstream to immune dysregulation or is simply a passive marker of disease. Prediagnostic autoantibody trends and temporal relationships help to address this question. Previously MPO-ANCA was detected both before anti-GBM antibody and anti-GBM diagnosis. We describe the natural history of subclinical ANCA in PLN and SLE without LN for the first time. Subclinical MPO-ANCA ≥ 3 U/mL was associated with PLN but not SLE without LN. A rising prediagnostic MPO-ANCA was also associated with future PLN. A prediagnostic MPO-ANCA that was both above 3 U/mL and continued to rise over time was most specific for developing PLN. These associations may be even stronger if the few SLE without LN disease controls with MPO-ANCA develop LN in the future. 3 U/mL is 50% of the upper limit of normal for the MPO-ANCA assay. But, abnormal ANCA levels were established in cohorts of patients with active vasculitis and not PLN or SLE without LN. It is reasonable that abnormal subclinical thresholds for other disease processes are different. Previous autoimmune disease studies have also established statistically significant prediagnostic thresholds within the normal diagnostic range consistent with these results.

Improved knowledge about subclinical MPO-ANCA trends could have prognostic implications. Overall, even low but significant MPO-ANCA may portend future PLN in SLE patients without LN. And up to 35% of SLE patients will manifest LN after initial diagnosis [31]. Closer follow-up in high risk MPO-ANCA positive SLE without LN patients could facilitate a more prompt diagnosis with earlier intervention to better preserve residual renal function. But, prospective confirmation in a cohort of SLE without LN is required before any formal clinical implementation.

Improved knowledge about subclinical MPO-ANCA trends could also broaden our understanding of PLN pathophysiology. There is a strong correlation between ANCA and dsDNAab at diagnosis, but the subclinical relationship was previously unknown [11, 17, 19]. We found that statistically significant subclinical MPO-ANCA levels preceded statistically significant subclinical dsDNAab levels (determined on the same samples and reported in a previous publication) when a clear antecedent antibody could be determined. And both autoantibodies are present before elevated CRP, a surrogate inflammatory marker for early subclinical disease. The summation of this data suggests that MPO-ANCA may participate upstream in the pathogenesis of PLN.

MPO-ANCA seropositivity has been previously attributed to cross reactive dsDNAab, but there is a body of evidence which suggests that this is not the only contribution [32, 33]. Jethwa et al. reported compelling in vitro evidence that DNA/dsDNAab complexes from active SLE patient sera can bind cationic MPO in ANCA immunoassays to cause a “false positive” test. However, disassociation of the DNA from the dsDNAab binding site reduced but did not normalize, subsequent MPO-ANCA levels to suggest that MPO-ANCA may indeed have been present. In addition, in our study, elevated dsDNAab was not associated with elevated MPO-ANCA levels. There were examples of profoundly elevated dsDNAab with nearly undetectable MPO-ANCA as well as significantly elevated MPO-ANCA with nearly undetectable dsDNAab. These findings do not support the theory that MPO-ANCA positivity is solely explained by dsDNAab cross reactivity. Cross reacting dsDNAab would result in a strong correlation between MPO-ANCA and dsDNA seropositivity and titer, because all assays were run on the same day on the same platform. Moreover, by evaluating preclinical quantitative MPO-ANCA, we were able to demonstrate statistically significant MPO-ANCA levels occurred before dsDNAab was present to bind MPO consistent with true antibody production. Finally, Falk et al. have identified the most pathogenic epitope specific MPO-ANCA (anti-MPO 1 antibody) which was elevated in 17% of the SLE disease controls [34, 35]. It is possible and likely that both real MPO-ANCA and DNA/dsDNAab bound MPO complexes may contribute to MPO-ANCA seropositivity.

MPO-ANCA stimulation of NETosis, the externalization of cellular chromatin and cytoplasmic protein containing fibers that capture pathogens or stimulate autoantibody production, is one intriguing hypothesis for MPO-ANCA directed pathogenicity. While NETosis has not been specifically evaluated previously, previous literature supports the hypothesis. MPO-ANCA triggers neutrophil NET deployment in vitro. There is increased NET formation in both MPO-ANCA vasculitis and SLE. NET burden can serve as a nidus for dsDNAab, ANA, C1q, and additional MPO-ANCA production to induce a deleterious positive feedback cycle. Both NET and autoantibody containing immune complexes can then cause end organ damage to include LN [36–41]. There is evidence that NETosis has a stronger association with LN than SLE without LN. Persistence of NET burden is associated with LN as well as elevated dsDNAab and anti-NET antibodies [37]. Netting neutrophils present in kidney biopsies are specifically associated with PLN and increased disease activity on kidney biopsy [40].

DoDSR study limitations inherent to the retrospective case-control design have been reported previously [23, 24]. For this specific study, the relatively low sample size limited power for subgroup analysis. Additional mesangial and membranous lupus nephritis disease controls were lacking. MPO-ANCA change over time calculations assume a linear rise which has not been proven. Absent comparison literature for preclinical MPO-ANCA evaluation below diagnostic thresholds was initially a concern. But, percent of healthy controls with MPO-ANCA ≥ 3 U/ml in this study was similar to that found in our anti-GBM disease study (23% versus 17%). And now we have reported significant differences between the percent of disease and control patients with subclinical biomarkers above thresholds within the normal diagnostic range in multiple studies [23, 24, 42, 43]. A final limitation is that small number of prediagnostic samples from PLN cases may have dated between SLE and PLN diagnosis. But, similar to the general population, the majority of PLN cases were diagnosed simultaneously or within a year of SLE diagnosis and when prediagnostic MPO-ANCA was positive, it was most often also positive in the earliest available sample. Because of these limitations, this data does not prove that MPO-ANCA directly contributes to the pathogenicity in a subpopulation of PLN. The data simply adds to our understanding of the complex and not fully elucidated pathophysiology of PLN.

Follow-up studies are required to more fully describe the subclinical pathophysiology of PLN. Preclinical presence, trajectory, and temporal relationship of anti-Smith, anti-RNP, anti-DNAase, anti-C1q, anti-lactoferin, anti-Cathepsin G, anti-elastase, and anti-NET antibody levels need to be evaluated in a larger cohort of PLN. Additional mesangial and membranous LN disease control comparison groups will strengthen analysis. Mesangial LN is a particularly important comparison group because it can have a similar clinical presentation to early PLN. In addition, we need to better characterize the prediagnostic MPO-ANCA to include epitope specificity, Fc glycosylation patterns, and avidity [34, 44–46]. Evaluation of the in vitro capability of PLN prediagnostic MPO-ANCA to trigger NET release from neutrophils would be needed to confirm pathogenic characteristics. MPO-ANCA may play a general role in autoimmune pathogenesis and at least partially explain observed subclinical and clinical autoimmune overlap syndromes.

MPO-ANCA may help delineate the SLE patients that are at risk for future PLN and may also directly contribute to the PLN pathogenesis. A more precise understanding of preclinical SLE pathogenesis may provide more specific future therapeutic targets for research.

## Figures and Tables

**Table:** (a) Proliferative lupus nephritis (PLN) background information on based on electronic medical record chart review. Medians presented with 25% and 75% values in parentheses for continuous data because they were not normally distributed. Some percentages are accompanied by *n*/*n* in parentheses. Prednisone dose was <10 mg/d. Other immunosuppression was discontinued at least 6 months before confirmatory kidney biopsy. Not all patients had information available for each laboratory measurement. SLE, systemic lupus erythematosus; PLN, progressive lupus nephritis; LN, lupus nephritis; CNS, central nervous system; RBC, red blood cell; dsDNA, double-stranded DNA; SM, smooth muscle; RNP, ribonucleoprotein; CRP, C-reactive protein; NIH, National Institutes of Health.

Average age (range)	36 years old (18–60)

Race	
Caucasian	15%
African American	60%
Other	25%

Gender	
Female	65%

History of HTN	83 (19/23)

History of DM	0 (0/23)

Arthralgia	70 (16/23)

Dermatologic	48 (11/23)

Hematologic	53 (12/23)

Cardiac involvement	35 (8/23)

CNS involvement	9 (2/23)

Lung involvement	35 (8/23)

Liver involvement	9 (2/23)

Hematuria (>3 RBC phf)	100 (23/23)

Proteinuria (>300 mg)	100 (23/23)

Nephrotic range proteinuria (>3.5 gm)	35 (8/23)

Proteinuria Quantification (average in gm)	2.36 (0.380–4.61)

Serum Creatinine (average mg/dl)	1.13 (0.6–2.4)

ANA (% positive)	96 (22/23)

dsDNA antibody (% positive)	82 (18/22)
Average (titer)	1 : 320
Average (U/mL peak; *n* = 6)	189 (130–228)

dsDNA antibody OR ANA (% positive)	100 (23/23)

ANCA (% positive)	0 (0/3)

Anti-phospholipid antibody (% positive)	38 (8/23)

Anti-SM antibody (% positive)	38 (6/16)

Anti-RNP antibody (% positive)	50 (8/16)

CRP	
(Average mg/dL)	2.1 (0.1–6.6)
(% >0.8 mg/dL)	79 (11/14)

Biopsy (%)	
WHO Class III	22 (5/23)
WHO Class IV	78 (18/23)
WHO Class IV + V	35 (8/23)
Crescent Formation	23 (6/23)

Median NIH activity index	8 (5,10)

Median NIH chronicity index	3 (1,3)

Median SLE disease activity index	16 (12,22)

Immunosuppression before biopsy (%)	
Prednisone	22 (5/23)
Hydroxychloroquine	39 (9/23)
Other (cyclophosphamide, mycophenolate mofetil, rituximab)	9 (2/23)

**Table:** (b) Systemic lupus erythematosus (SLE) without lupus nephritis (LN) disease control background information on based on ICD-9 codes provided by the Department of Defense Serum Repository (DoDSR). Laboratory data were not available for these disease control patients.

Average age (range)	36 years old (18–60)

Race	
Caucasian	15%
African American	60%
Other	25%

Gender	
Female	65%

History of HTN	36%

History of DM	9%

Renal involvement	0%

Arthralgia	73%

Dermatologic	46%

Hematologic	41%

Anti-phospholipid positive	23%

Cardiac involvement	9%

CNS involvement	9%

Lung involvement	18%

Liver involvement	5%

Median SLE disease activity index	10 (4,14)

**(a):** 

MPO-ANCA	Cases (%)	Healthy controls (%)	OR (odds ratio)	CI (confidence interval)	*p* value (Fisher's exact)
(≥3 U/mL):					
All	91 (21/23)	26 (6/23)	30	5.3–167	<0.001
<1 year	88 (15/17)	19 (3/16)	33	4.7–226	<0.001
1–4 years	87 (13/15)	13 (2/15)	42	5.2–347	<0.001
>4 years	69 (11/16)	25 (4/16)	6.6	1.4–31	0.03
(≥6 U/mL)					
All	57 (13/23)	0 (0/23)	34^*∗*^	3.9–300^*∗*^	<0.001
<1 year	59 (10/17)	0 (0/16)	28^*∗*^	2.9–262^*∗*^	<0.001
1–4 years	7 (1/15)	0 (0/15)	1.1^*∗*^	0.1–20^*∗*^	1.0
>4 years	19 (3/16)	0 (0/16)	3.9^*∗*^	0.4–42^*∗*^	0.23

**(b):** 

MPO-ANCA	Cases (%)	Disease controls (%)	OR (odds ratio)	CI (confidence interval)	*p* value (Fisher's exact)
(≥3 U/mL):					
All	91 (21/23)	43 (9/21)	14	2.6–76	<0.001
<1 year	88 (15/17)	39 (5/13)	12	1.9–76	0.007
1–4 years	87 (13/15)	38 (6/16)	11	1.8–66	0.009
>4 years	69 (11/16)	44 (7/16)	2.8	0.7–12	0.29
(≥6 U/mL)					
All	57 (13/23)	5 (1/21)	26	3.0–228	<0.001
<1 year	59 (10/17)	8 (1/13)	17	1.8–164	0.006
1–4 years	7 (1/15)	6 (1/16)	1.1	0.1–19	1.0
>4 years	19 (3/16)	0 (0/16)	4.9^*∗*^	0.5–50^*∗*^	0.23

**Table 3:** Conditional logistic regression (CLR) for PLN MPO-ANCA by unit versus healthy controls, SLE without LN disease controls, and all controls together.

CLR for MPO-ANCA by unit: PLN versus controls	OR (odds ratio)	CI (confidence interval)	*p* value
Healthy controls	9.1	3.4–24.5	<0.001
Disease controls	1.5	1.2–1.8	<0.001
All controls	7.1	1.2–44.2	<0.001

**(a):** 

Change in MPO-ANCA (U/mL/year)	Cases (%)	Healthy controls (%)	OR (odds ratio)	CI (confidence interval)	*p* value (Fisher's exact)
>0 U/mL	70 (14/20)	10 (2/20)	21	3.7–120	<0.001
>0.3 U/mL	60 (12/20)	0 (0/20)	32^*∗*^	3.6–291^*∗*^	<0.001
>0.5 U/mL	45 (9/20)	0 (0/20)	18^*∗*^	2.1–161^*∗*^	0.001
>1 U/mL	20 (4/20)	0 (0/20)	6.3^*∗*^	0.7–59^*∗*^	0.10

**(b):** 

Absolute rise in MPO-ANCA (U/mL)	Cases (%)	Healthy controls (%)	OR (odds ratio)	CI (confidence interval)	*p* value (Fisher's exact)
>0 U/mL	70 (14/20)	10 (2/20)	21	3.7–120	<0.001
>1 U/mL	55 (11/20)	0 (0/20)	27^*∗*^	3.0–237^*∗*^	<0.001
>2 U/mL	35 (7/20)	0 (0/20)	12^*∗*^	1.4–110^*∗*^	0.008
>3 U/mL	30 (6/20)	0 (0/20)	10^*∗*^	1.1–91^*∗*^	0.02

**(c):** 

Change in MPO-ANCA (U/mL/year)	Cases (%)	Disease controls (%)	OR (odds ratio)	CI (confidence interval)	*p* value (Fisher's exact)
>0 U/mL	70 (14/20)	16 (3/19)	12	2.6–59	0.001
>0.3 U/mL	60 (12/20)	5 (1/19)	27	3.0–49	<0.001
>0.5 U/mL	45 (9/20)	0 (0/19)	20^*∗*^	2.2–179	0.001
>1 U/mL	20 (4/20)	0 (0/19)	5^*∗*^	0.5–49	0.10

**(d):** 

Absolute rise in MPO-ANCA (U/mL)	Cases (%)	Disease controls (%)	OR (odds ratio)	CI (confidence interval)	*p* value (Fisher's exact)
>0 U/mL	70 (14/20)	16 (3/19)	12	2.6–59	0.001
>1 U/mL	55 (11/20)	11 (2/19)	10	1.9–57	0.006
>2 U/mL	35 (7/20)	5 (1/19)	10	1.1–89	0.04
>3 U/mL	30 (6/20)	0 (0/30)	10^*∗*^	1.1–93^*∗*^	0.02

**Table 5:** Temporal relationship of detectable autoantibodies. Patients without a clear antecedent antibody above the designated threshold were not included in analysis. OR, odds ratio; CI, confidence interval.

Comparison thresholds	Antecedent MPO-ANCA (%)	Antecedent dsDNAab (%)	OR	CI	*p* value (Fisher's exact)
MPO-ANCA ≥ 3U/ml versus dsDNAab ≥ 3 IU/ml	89 (8/9)	11 (1/9)	64	3.4–1211	0.003

MPO-ANCA ≥ 3 U/ml versus dsDNAab ≥ 5 IU/ml	100 (10/10)	0 (0/10)	100^*∗*^	5.5–1831^*∗*^	<0.001

## Supplementary Materials

Supplementary Materials Supplemental Table  1: a comparison of the percent of PLN cases with an elevated dsDNAab that have a concurrent elevated MPO-ANCA versus those that have a normal MPO-ANCA.
