# Supplementary material for: Anti-Myeloperoxidase Antibodies Associate with Future Proliferative Lupus Nephritis
Source: Autoimmune Dis. 2017 Dec 24;2017:1872846. doi: 10.1155/2017/1872846 (PMC5757094; doi:10.1155/2017/1872846)
Supplement: Supplementary Materials — Supplemental Table 1: a comparison of the percent of PLN cases with an elevated dsDNAab that have a concurrent elevated MPO-ANCA versus those that have a normal MPO-ANCA. [file 1872846.f1.docx]

|  | MPO-ANCA (≥6 U/ml) | MPO-ANCA (≤6 U/ml) | P-Value |
| --- | --- | --- | --- |
| dsDNA ≥10 IU/ml | 40 | 60 | 0.11 |
| dsDNA ≥20 IU/ml | 56 | 44 | 0.58 |
| dsDNA ≥40 IU/ml | 58 | 42 | 0.38 |
| dsDNA ≥100 IU/ml | 60 | 40 | 0.46 |

Supplemental Table 1:

A comparison of the percent of PLN cases with an elevated dsDNAab that have a concurrent elevated MPO-ANCA versus those that have a normal MPO-ANCA.
